# High costs of infection: Alphavirus infection reduces digestive function and bone and feather growth in nestling house sparrows (*Passer domesticus*)

**DOI:** 10.1371/journal.pone.0195467

**Published:** 2018-04-06

**Authors:** Carol A. Fassbinder-Orth, Tess L. Killpack, Dylan S. Goto, Ellecia L. Rainwater, Valerie I. Shearn-Bochsler

**Affiliations:** 1 Biology Department, Creighton University, Omaha, NE, United States of America; 2 Biology Department, Salem State University, Salem, MA, United States of America; 3 School of Medicine, Creighton University, Omaha, NE, United States of America; 4 USGS National Wildlife Health Center, Madison, WI, United States of America; Keele University Faculty of Natural Sciences, UNITED KINGDOM

## Abstract

Increasingly, ecoimmunology studies aim to use relevant pathogen exposure to examine the impacts of infection on physiological processes in wild animals. Alphaviruses are arthropod-borne, single-stranded RNA (ssRNA) viruses (“arboviruses”) responsible for millions of cases of human illnesses each year. Buggy Creek virus (BCRV) is a unique alphavirus that is transmitted by a cimicid insect, the swallow bug, and is amplified in two avian species: the house sparrow (*Passer domesticus*) and the cliff swallow (*Petrochelidon pyrrhonota)*. BCRV, like many alphaviruses, exhibits age-dependent susceptibility where the young are most susceptible to developing disease and exhibit a high mortality rate. However, alphavirus disease etiology in nestling birds is unknown. In this study, we infected nestling house sparrows with Buggy Creek virus and measured virological, pathological, growth, and digestive parameters following infection. Buggy Creek virus caused severe encephalitis in all infected nestlings, and the peak viral concentration in brain tissue was over 34 times greater than any other tissue. Growth, tissue development, and digestive function were all significantly impaired during BCRV infection. However, based on histopathological analysis performed, this impairment does not appear to be the result of direct tissue damage by the virus, but likely caused by encephalitis and neuronal invasion and impairment of the central nervous system. This is the first study to examine the course of alphavirus diseases in nestling birds and these results will improve our understanding of age-dependent infections of alphaviruses in vertebrate hosts.

## Introduction

Interest in the interplay among infectious diseases, their associated immune responses, and life history traits in wild organisms has dramatically increased over the past two decades [1, 2, 3]. Life history traits commonly examined in the context of disease and immune function include reproductive success, growth, and physiological functions like digestion and absorption of nutrients [4]. However, disease severity, immunocompetence, and physiological functions can be difficult to measure on a whole-organismal level. Many previous studies of trade-offs between investment in the immune system and other physiological processes often have used immunological stimuli such as lipopolysaccharide (LPS), keyhole limpet hemocyanin (KLH), or phytohemmaglutinin (PHA) to measure the impacts on various physiological functions, but these stimuli are limited in their ability to mimic the true complexity of immunological encounters that an organism faces during pathogenic exposure [3, 5, 6, 7, 8, 9]. Increasingly, studies examining the impacts of an infection on physiological processes in wild animals are being performed (e.g. [10, 11]). Wild birds have been common test subjects for studies in ecoimmunology and disease ecology, often due to their accessibility, ease of study and sample collection, and diversity of life history strategies. Birds also serve as hosts for many arboviruses. Arthropod-borne viruses (Arboviruses) are one of the primary causes of acute neurological disease in vertebrates, with alphaviruses being one of the most predominant neuroinvasive arboviruses [12].

Alphaviruses (family *Togaviridae*) are globally distributed, single stranded RNA arboviruses, with approximately 30 different alphaviruses identified [13]. The frequency of alphavirus epidemics has increased in recent years, likely due to increases in urbanization and travel [14]. A unique and understudied new world member of the western equine encephalitis complex is Buggy Creek virus (BCRV). BCRV, like many other alphaviruses, has avian reservoir hosts. However, BCRV differs significantly from other alphaviruses because it is vectored primarily by an ectoparasitic swallow bug (Hemiptera: Cimicidae: *Oeciacus vicarius*), rather than by mosquitoes. Swallow bugs are insects in the same family as the common bed bug (*Cimex lectularius*). However, unlike the bedbug, which is not known to be a competent vector for any pathogens [15], the swallow bug is a replicative host for BCRV and the only known competent vector for the virus [16].

Swallow bugs closely associate with the colonially nesting cliff swallow (*Petrochelidon pyrrhonota*) and the introduced house sparrow (*Passer domesticus*) that occupies nests in cliff swallow colonies. The BCRV host-pathogen system is intriguing because it is one in which a native avian host (the cliff swallow) is apparently more resistant than a non-native invasive bird (the house sparrow) to viral infection and illness [17, 18]. However, it is entirely unknown what factors contribute to the documented variability in disease susceptibility for the avian hosts in the BCRV system or in other arbovirus systems (*e*.*g*., West Nile virus). Previous studies of avian arbovirus infections have largely been limited to virological and serological studies [19, 20, 21, 22, 23], and descriptions of pathological damage of field caught birds [18]. Such measures do not provide a comprehensive assessment of the physiological responses during infection of the avian hosts.

Systemic inflammation triggered by the innate immune system is important for an effective antiviral response; however, high activity of pro-inflammatory immune responses can cause collateral tissue damage in animals [24, 25]. Severe alphavirus-induced disease is characterized by high levels of viral replication and overproduction of cytokines (“cytokine storm”) and is often found in young animals [26, 27]. This pattern of age-dependent disease susceptibility was first reported for human alphavirus infections more than 50 years ago [28]. Since then, the majority of studies performed on age-dependent alphavirus susceptibility have been experimental alphavirus infections of mice or clinical reports of alphavirus infections in humans [29, 30, 31, 32]. Nestling birds have been shown to be more susceptible than adults to several arboviruses, including flaviviruses and alphaviruses [18, 33, 34, 35, 36], though the specific explanatory mechanisms are lacking.

The incidence of naturally-acquired BCRV infection levels are low in nestling cliff swallows (~2%) but higher in nestling house sparrows (20%) [18]. There is no record of BCRV-induced disease in wild-caught nestling or adult cliff swallows or adult house sparrows. Experimental inoculation of adult house sparrows with BCRV causes minimal viremia, no recorded mortality, and significant upregulation of pattern recognition receptors and cytokines associated with antiviral and pro-inflammatory responses [3]. Severe BCRV-induced neurological disease and high levels of viral load have been recorded in wild-caught house sparrow nestlings [18] Furthermore, house sparrow nestlings have been shown to have immature innate and adaptive immune systems [37, 38], which could lead to inefficient immune response and regulation, and increased BCRV disease susceptibility. BCRV infection in nestling house sparrows appears to have broad ecological consequences. Among field-sampled nestling house sparrows tested at 4–6 days of age, only 12.8% that were positive for BCRV ultimately fledged, versus 50.5% of those that were negative [39]. However, the mechanism by which BCRV infection impairs fledging success is unknown.

The nestling period of birds is energetically expensive due to the rapid anatomical growth and maturation that occurs during this time. Inflammatory responses to infection can be costly to birds in terms of energy, pathological damage, decreased appetite and impaired growth [40, 41]. Impaired nestling growth has been shown to delay fledging [42, 43, 44], and decrease predicted survival of post-fledging birds in the wild [45, 46]. Therefore, the impacts of infectious diseases may influence the success of the nestling and fledgling periods of birds. Nestling development is closely linked to digestive function, and the time course for development of the digestive system and body size and composition in altricial nestlings has been documented under normal conditions and in conditions of altered food availability and quality [47, 48]. However, to our knowledge, no studies exist which have examined the impacts of an active pathogen infection on the development and physiology of nestling birds. In this study, we infected house sparrow nestlings with BCRV, quantified infection severity through serological methods and pathology, and measured the impacts of infection on digestive function, bone growth, and feather growth.

## Materials and methods

### Ethics statement

All procedures involving animals were approved by the Creighton University Institutional Animal Care and Use Committee under protocol 0932. Animals were euthanized by CO_2_ overdose, followed by cervical dislocation. House sparrows are a non-native species, and federal and state permits are not required for collection of this species. House sparrows were collected from private land, and landowner permission to collect the nestlings was granted prior to start of the experiment.

### Study organisms

Three or four day-old house sparrow (*Passer domesticus*) nestlings were collected May-June, 2012, from nest boxes in the Council Bluffs, Iowa, and Omaha, Nebraska area. Nestlings were transported to the laboratory and housed in a plastic beaker lined with a waterproof mat in a temperature-controlled room (35°C) and 15-hour illumination (equivalent to the natural photoperiod during May-June in our study area).

Nestlings were weighed before the first feeding at 06:30h and after the last feeding at 20:30h. Skeletal lengths of the head (skull + beak) and tarsus (notch of the intertarsal joint to the tarsometatarsal bone were measured daily with a digital caliper to the nearest 0.10 mm [48]. A digital caliper was also used to measure wing chord length of birds (bend of the wing to the tip of the longest primary feather) post-mortem.

Nestlings were forced-fed a moistened protein-enriched diet, as described in Lepczyk et al. [47] through blunt end syringes fifteen times a day. The mass per feeding was determined by previous knowledge determining the age-dependent energy needs of house sparrows [49, 50] and all nestlings were force-fed the age-specific meal size regardless of treatment.

### Experimental design

House sparrow nestlings were randomly assigned to one of two treatment groups (BCRV or Control), and nestlings from the same brood were randomly assigned different treatments groups. At 7 days of age, nestlings were inoculated subcutaneously on the breast with 75 μl containing 3 x 10^3^ plaque forming units (PFU) of BCRV (lineage A, isolate H255A), or a buffered saline solution (Control) [n = 12 nestlings / treatment group]. The course of the infection was monitored for 4 days post inoculation (DPI), with four nestlings from each treatment euthanized at 2 DPI, 3 DPI, and 4 DPI. Nestlings were not kept past 4 DPI because BCRV-infected birds were unable to remain upright or swallow by 4 DPI and were euthanized for humane reasons no later than 4 DPI.

### Sample collection and processing

#### Blood

Blood samples were collected daily from each nestling 0–4 DPI via jugular or brachial venipuncture. Blood samples were placed in serum separator tubes containing complete Dulbecco’s Modified Eagle Media (DMEM), containing 10% fetal bovine serum, penicillin (100 U/ml), streptomycin (100μg/ml), and amphotericin B (0.25 μg/ml). Tubes were placed on wet ice, centrifuged, and then stored at -80°C.

#### Oral swabs

The inner beak, oral cavity, and throat were swabbed with sterile, Dacron swabs daily post inoculation. The swab was placed into a tube containing 1 ml of complete DMEM media, placed on wet ice, and later transferred to storage at -80°C.

#### Tissues

Four nestlings from each treatment group were euthanized by CO_2_ overdose followed by cervical dislocation on 2 DPI, 3 DPI, and 4 DPI. Necropsies were performed, the gut was removed from each nestling, flushed with saline, measured to the nearest 0.1 centimeter, and blotted dry before being weighed. Subsamples of the skin, skeletal muscle, lung, heart, liver, small intestine, pancreas, spleen, bursa, kidney, cerebrum, and cerebellum were immediately frozen in liquid nitrogen from each nestling for future virological analysis. Subsamples of all tissues were also collected for pathology. Remaining intestinal tissue was flushed with cold PBS (pH 7.4), cut into proximal, medial, and distal sections, and then blotted dry before recording the mass and length of each section. Intestinal sections, as well as pancreas samples, were then stored at -80°C until further analysis for digestive enzyme activity. Remaining muscle and liver samples were stored at -80°C until further analysis for tissue maturation.

### Plaque assay for analysis of viral loads

Levels of cytopathic BCRV were assessed in blood, swab, and tissue samples using a Vero plaque assay, according to [51]. Briefly, Vero cells in complete DMEM media were plated onto each well of a six-well plate and incubated for 3–4 days at 37°C and 5% CO_2_. Diluted samples were added to each well and incubated for 45 min. After incubation, 3 mL of an agar overlay was added for plaque visualization. Plates were incubated at 37°C and 5% CO_2_ and checked for cytopathic effect at 72 h and 96 h. The number of plaques observed was recorded and virus concentrations were reported as PFU/ ml (blood, oral swabs) or PFU/ mg (tissues).

### Pathology

Tissues were placed in 10% buffered formalin for two weeks, then they were trimmed, embedded in paraffin, sectioned at 5 μm, and stained with hematoxylin and eosin. Slides were then examined and blindly scored for histopathology at the USGS National Wildlife Health Center (Madison, WI).

### Tissue maturation

Liver and muscle (flight + leg) samples were further analyzed for tissue composition. Wet masses of the liver and muscle samples were recorded (±0.10 mg) and dried to constant mass at 65°C. Dry liver and muscle masses were recorded, water mass (g wet tissue-g dry tissue) was calculated, and dried liver and muscle samples were individually wrapped in pre-dried filter paper for lipid extraction in diethyl ether in a modified Soxhlet side-arm extractor. Lean dry mass and tissue mass extracted (g dry tissue-g lean dry tissue), as well as indices of muscle water content (g water/g lean dry tissue) and liver lipid content (g tissue extracted/g lean dry tissue) were calculated.

### Digestive efficiency and excreta lipid content

Excreta was collected from each nestling starting 24 hours after arrival to the Animal Care Facility to allow for elimination of previously consumed food from the animals’ digestive systems. Excreta was collected at hourly feedings directly from the bird or removed from its nest container. Excreta was then dried for 48 to 96 hours at 50°C, weighed, then stored at -20°C until further analysis. Dry excreta samples were used in digestive efficiency calculations and used to calculate excreta lipid content.

Digestive efficiency was measured according to [52]. Briefly, subsamples of food were dried for 24 hrs at 60°C, and the daily mass intake was determined as wet mass ingested x percentage dry mass. Because endogenous wastes mix with undigested material in the cloaca, the assimilable mass coefficient (AMC; a measure of assimilation efficiency) is an underestimate of the true assimilable mass coefficient [53]. Therefore, we express digestive efficiency as an apparent AMC, which is calculated as (Qi −Qe)/Qi, where Qi = the rate of food intake (g of daily mass (DM)/d); and Qe = the rate of excreta production (g of DM/d; [54]).

For lipid content analysis, lipids were extracted from the dry excreta samples using diethyl ether in a modified Soxhlet side-arm extractor. Sample packets were dried again for 24 hours and then weighed to determine the percent lipid extracted.

### Digestive enzymes

Intestinal brush border enzyme activity was assessed in whole tissue homogenates of the proximal, medial, and distal intestinal sections. Maltase and aminopeptidase-N were measured because they index the majority of brush border carbohydrate and peptide hydrolysis, respectively. Prior to analysis, samples were thawed and homogenized with cold 300 mM mannitol in 1 mM HEPES-KOH buffer (pH 7.5). Maltase activities were measured according to Dahlqvist [55] as modified by Martınez del Rio [56]. Briefly, gut homogenates were incubated with 56 mM maltose in 0.1 M maleate and NaOH buffer, pH 6.5, at 40°C for 20 min. Next, stop-develop reagent (glucose assay kit; Sigma-Aldrich, St. Louis, MO) was added to each tube, vortexed, and incubated at 40°C for 30 min. Lastly, 12 N H_2_SO_4_ was added to each tube, and the absorbance was read at 540 nm. Activity was normalized by intestinal wet mass.

Aminopeptidase-N activity was measured according to [50], with some modifications. Briefly, diluted gut homogenate was added to assay mix (2.0 mM L-alanine p-nitroanilide in 1 part 0.2 M NaH_2_PO_4_-Na_2_HPO_4_, pH 7.0, and 1 part deionized H_2_O) previously heated to 40°C. The reaction solution was incubated for 25 min at 40°C, ice cold 2 N acetic acid was added, and absorbance was measured at 384 nm. Apparent Michaelis constant (Km) and optimal pH for intestinal aminopeptidase-N activity were 1.1 ± 0.2mM (mean ± SE, n = 6) and 7.5, respectively. Activity was normalized per gram of intestinal wet mass.

To quantify pancreatic lipase activity, Quantichrom Lipase Assay Kit (BioAssay Systems, Hayward, CA) was used according to manufacturer’s recommendations and the absorbance was read at 412 nm using a microplate reader.

To quantify pancreatic amylase activity, aliquots of pancreas homogenate (diluted in 20 mM of sodium phosphate buffer with 6.7 mM NaCl pH 6.9) were added to each tube. Next, 1% potato starch was added and incubated at 40°C for 3 minutes. The reaction was stopped upon the addition of amylase color reagent to each tube. The tubes were placed in boiling water for 10 minutes and then cooled on ice for 3 minutes. Next, distilled water was added to the samples and the samples were read at 540 nm using a microplate reader.

To quantify pancreatic trypsin and chymotrypsin activity, pancreas samples were homogenized in 0.5 M Tris/HCl (pH 8.2) with 0.2 M CaCl_2_. Enterokinase (0.3%) was added to each sample and incubated for 1 hour at room temperature. Next, 1 mM BAPNA (N-alpha-benzoyl-*dl*-arginine-p-nitroanilide, pH 8.2) or GAPNA (N-glutaryl-l-phenylalanine-p-nitroanilide, pH 7.6) was added as the substrate for trypsin and chymotrypsin, respectively, and incubated for 10 minutes at 40°C. Next, 30% acetic acid was added to each tube for termination of the reaction and read at 410 nm using a microplate reader.

### Statistical analyses

JMP Pro 12 was used for all statistical analyses. A comparison of 2 DPI viral loads across tissues was performed using a linear mixed effect model with bird as a random effect. Comparisons of 3 DPI and 4 DPI viral loads across all tissues were not run due to low sample-sizes and non-normality of the data. Viral load in the cerebrum and lungs were analyzed for differences over time from 2 DPI to 4 DPI using one-way ANOVA. Analyses of viral load over time in the remaining tissues (heart, kidney, liver, muscle, skin, small intestine) were not run due to low sample sizes and non-normality of the data. Viral load data were log-transformed for analyses. Measures of body mass, head-beak length, tarsus length, AMC, and excreta lipid content from live control and BCRV-inoculated birds were compared by two-sample t-test at each day post inoculation (0 DPI, 1 DPI, 2 DPI, and 3 DPI). Data from 4 DPI are presented in figures but were not compared statistically due to low 4 DPI cohort sample size (n = 4 per group). Post-mortem measures of liver lipid content, muscle water content, wing chord, and digestive enzymes (pancreatic and intestinal) were compared by two-way ANOVA with infection treatment and DPI as factors. There were no significant interactions for any measure; interactions were dropped from the models before testing for fixed effects. In all tests, the significance level was set at α = 0.05.

## Results

### Viral detection

Viremia was quantified using a Vero plaque forming assay (N = 12/treatment). No viremia was detected in the blood in any group at 0 DPI. BCRV was detected in the BCRV-infected group on 1 and 2 DPI, with peak viremia levels occurring at 1 DPI in BCRV-infected birds (1.7 x 10^4^ ± 4.4 x 10^4^ PFU/ml blood), and declining to 5.8 x 10^3^ ± 2.8 x 10^3^ PFU/ml blood by 2 DPI (data not shown). No viremia was detected in the blood on 3 or 4 DPI. Virus was detected in oral swabs of one bird on 1 DPI (1 PFU/ml swab fluid) and one bird on 4 DPI (2 PFU/ml swab fluid, data not shown).

The viral load of 8 different tissues (cerebrum, heart, kidney, liver, lung, small intestine, skin, and skeletal muscle) was determined post mortem for all birds 2–4 DPI (Table 1). BCRV was detected in all tissue types analyzed at 2 DPI and viral loads differed among tissues (F_7,21_ = 61.28, p<0.0001). Viral load in the cerebrum was significantly higher than all other tissues at 2 DPI and load in the lungs was significantly higher than all tissues except the cerebrum (Tukey’s p<0.001 for all significant pairwise comparisons). Viral load decreased from 2 DPI to 4 DPI in the cerebral tissues (F_2,9_ = 5.01, p = 0.035, Tukey p = 0.049 comparing 2 DPI and 4 DPI) and the lung tissues (F_2,9_ = 5.35, p = 0.030, Tukey p = 0.024 comparing 2 DPI and 4 DPI).

**Table 1:** Viral load (mean ± SEM PFU BCRV/mg tissue) in tissues of BCRV-infected birds. N = 4 per sample type and DPI.

Sample type	DPI	Viral load (Mean ± SEM)	Percent individuals positive for BCRV
Cerebrum	2	7993±2729	100
	3	5250±272	100
	4	1503±840	100
Heart	2	1	50
	3	0	0
	4	1.26	25
Kidney	2	3±1.25	50
	3	1	50
	4	1±0.12	50
Liver	2	3±1.11	75
	3	0	0
	4	0	0
Lung	2	235±132	100
	3	26±16	100
	4	7±4	100
Small Intestine	2	3±0.58	75
	3	10±3.5	50
	4	5±1.25	50
Skeletal Muscle	2	1.5±0.25	75
	3	0	0
	4	0	0
Skin	2	1.26	25
	3	1.26	25
	4	0	0

### Pathology

**Fig 1:**
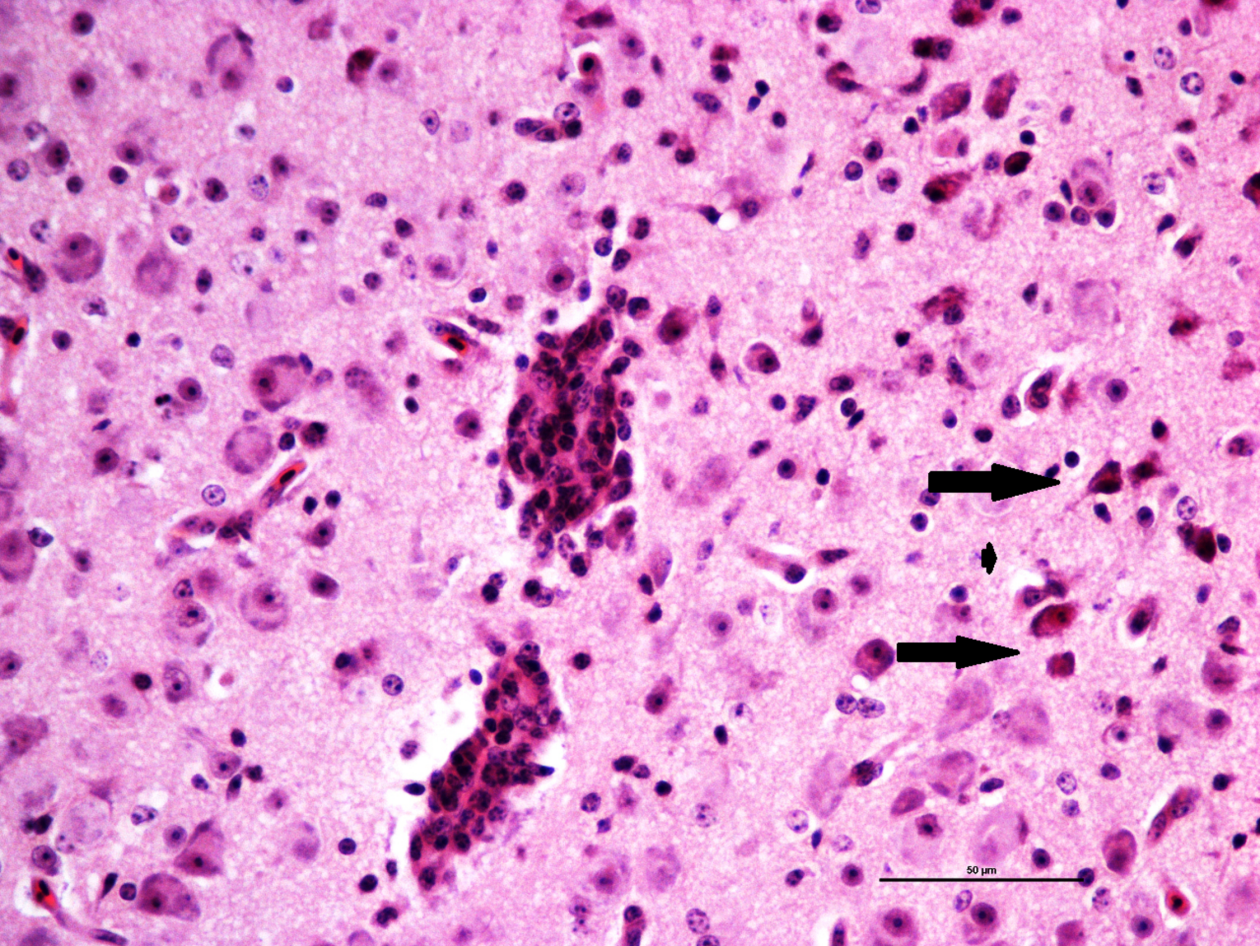
Brain sample of a nestling house sparrow infected with BCRV. Mild lymphoplasmacytic cuffing and perivascular edema, cerebrum. Several shrunken, deeply eosinophilic, necrotic neurons are present in the vicinity of the affected blood vessel (arrows).

Histopathologic abnormalities in BCRV infected birds were confined to the brain and meninges. All treatment birds developed mild to moderate, multifocal lymphoplamacytic and heterophilic encephalitis, as well as mild lymphoplasmacytic meningitis. These findings were most marked in the cerebrum and optic lobe, with the cerebellum and brainstem less affected. Encephalitis was characterized by multifocal lymphoplasmacytic cuffing (1–5 cell layers), perivascular edema, infiltration of the neuropil by heterophils, lymphocytes, macrophages, gliosis, and multiple necrotic neurons (Fig 1).

The most striking change in the cerebellum was edema of the Purkinje cell layer with occasional necrotic Purkinje cells. Gliosis and rarely, lymphoplasmacytic cuffing were present in the molecular layer of the cerebellum.

No significant abnormalities were seen in the kidneys, heart, skin, liver, skeletal muscle, spleen, bursa of Fabricius, intestine, or ventriculus in control or BCRV-inoculated nestlings. No abnormalities were present in the brain or meninges of control nestlings.

### Growth and maturation

**Fig 2:**
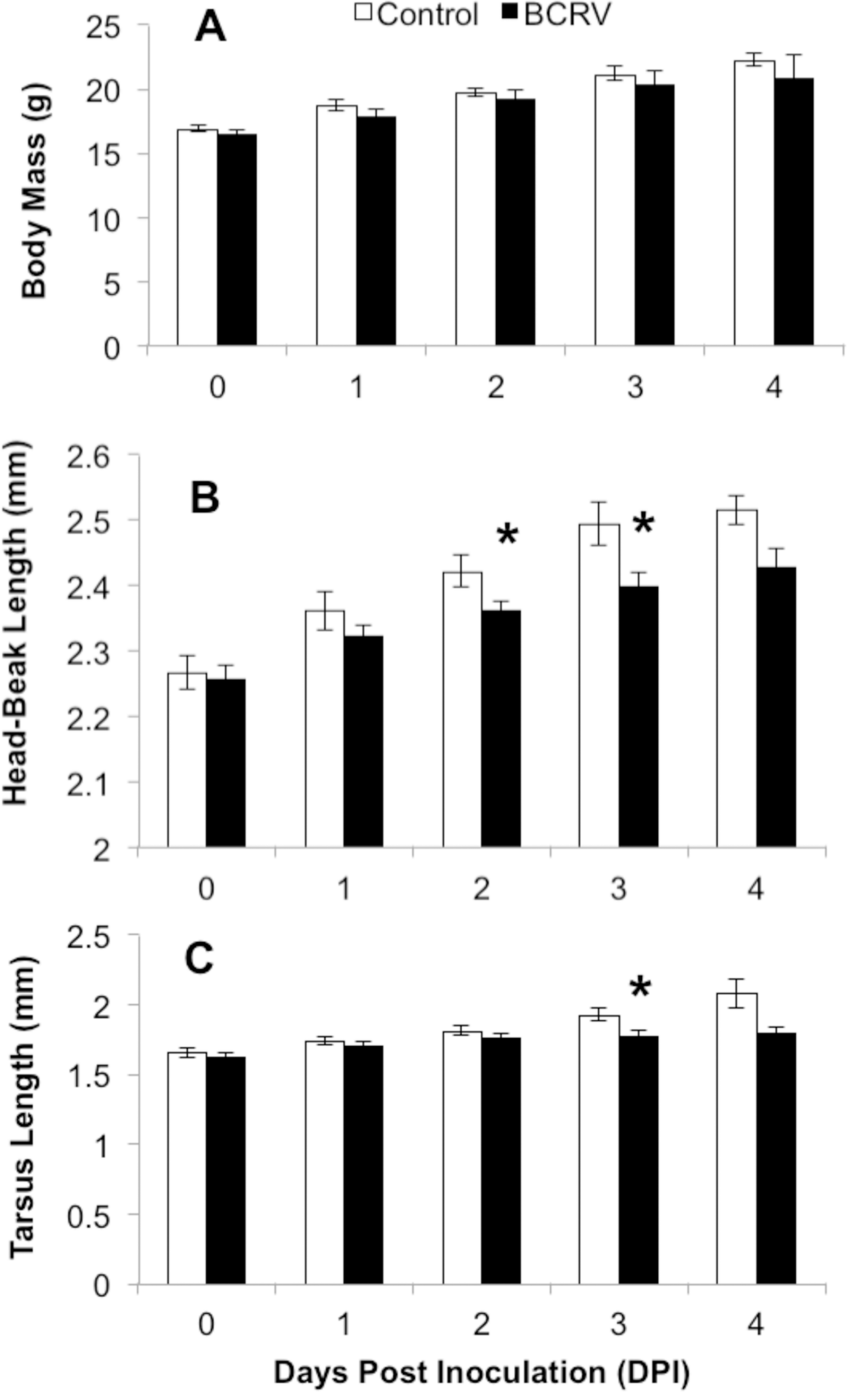
**Daily measures of (A) body mass, (B) head-beak and (C) tarsus lengths during infection (mean ± SEM)**. Nestlings were age 7 days post-hatch when infected (0 DPI). n = 11 at 0-2DPI; n = 7–8 at 3DPI, n = 4–5 at 4 DPI. Asterisks above bars indicate significant difference between treatment groups by two-sample t-test (4 DPI was not analyzed due to low sample size).

Body mass and skeletal lengths were measured on live birds following inoculation. There were no significant differences in body mass between control and treatment groups at any day post inoculation (Fig 2A; 0DPI t_20_ = 1.09, p = 0.289; 1DPI t_20_ = 1.47, p = 0.157; 2DPI t_20_ = 0.67, p = 0.508; 3DPI t_13_ = 0.69, p = 0.501), likely due to the forced feeding protocol that was maintained throughout the experiment. However, BCRV-inoculated nestlings did show decreased skeletal development after inoculation, despite uniform feed consumption rates compared to controls. Head-beak length of BCRV- inoculated nestlings was significantly smaller than non-infected controls at 2 DPI (t_20_ = 2.27, p = 0.034) and 3DPI (t_13_ = 2.55, p = 0.024) (Fig 2B), and tarsus length was significantly smaller than non-infected controls at 3 DPI (t_13_ = 2.92, p = 0.012) (Fig 2C).

**Fig 3:**
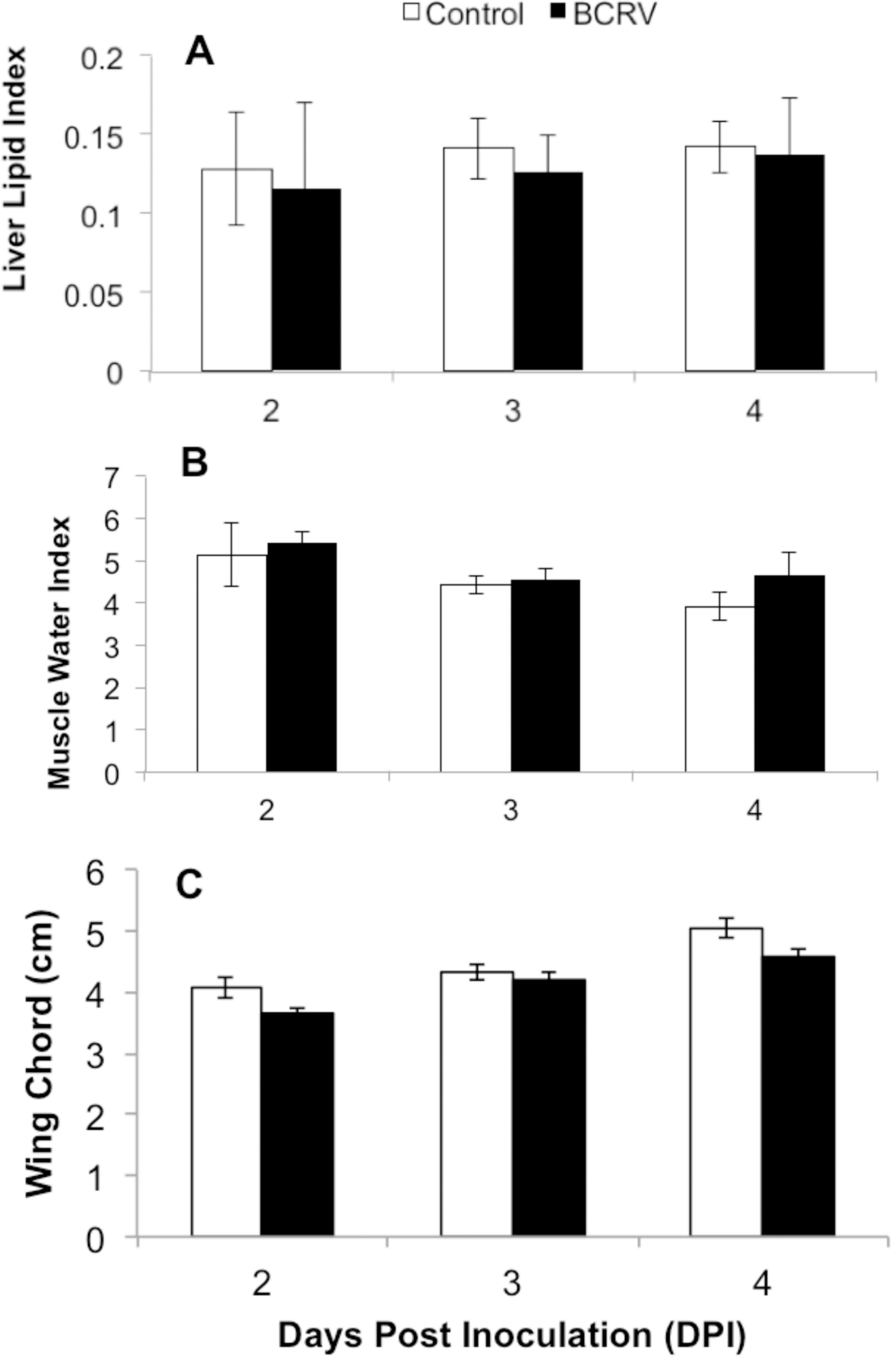
**Liver lipid index (A), muscle water index (B), and wing chord length (C) during infection (mean ± SEM).** Nestlings were age 7 days post-hatch when infected (0 DPI). n = 4 per group. Data were analyzed by two-way ANOVA.

Measures were taken from cohorts of birds post-mortem at 2, 3, and 4 DPI (n = 4 per group) to examine the impacts of infection on tissue maturation and feather growth. There were no significant interactions between DPI and treatment group for any measure. Liver lipid index was significantly lower in BCRV-infected birds compared with controls (Treatment F = 5.71, p = 0.027; DPI F = 0.35, p = 0.710) (Fig 3A). Muscle water index, a measure of functional immaturity of the tissue, did not significantly differ between BCRV-infected and control individuals, but significantly decreased with DPI as expected given the increasing age of the birds (Treatment F = 1.85, p = 0.189; DPI F = 4.45, p = 0.025) (Fig 3B). Wing chord (length of the wing feathers) significantly increased with DPI (DPI F = 31.41, p<0.001), and was significantly shorter in BCRV-infected birds compared with controls (Treatment F = 11.54, p = 0.003) (Fig 3C).

### Digestive function

**Fig 4:**
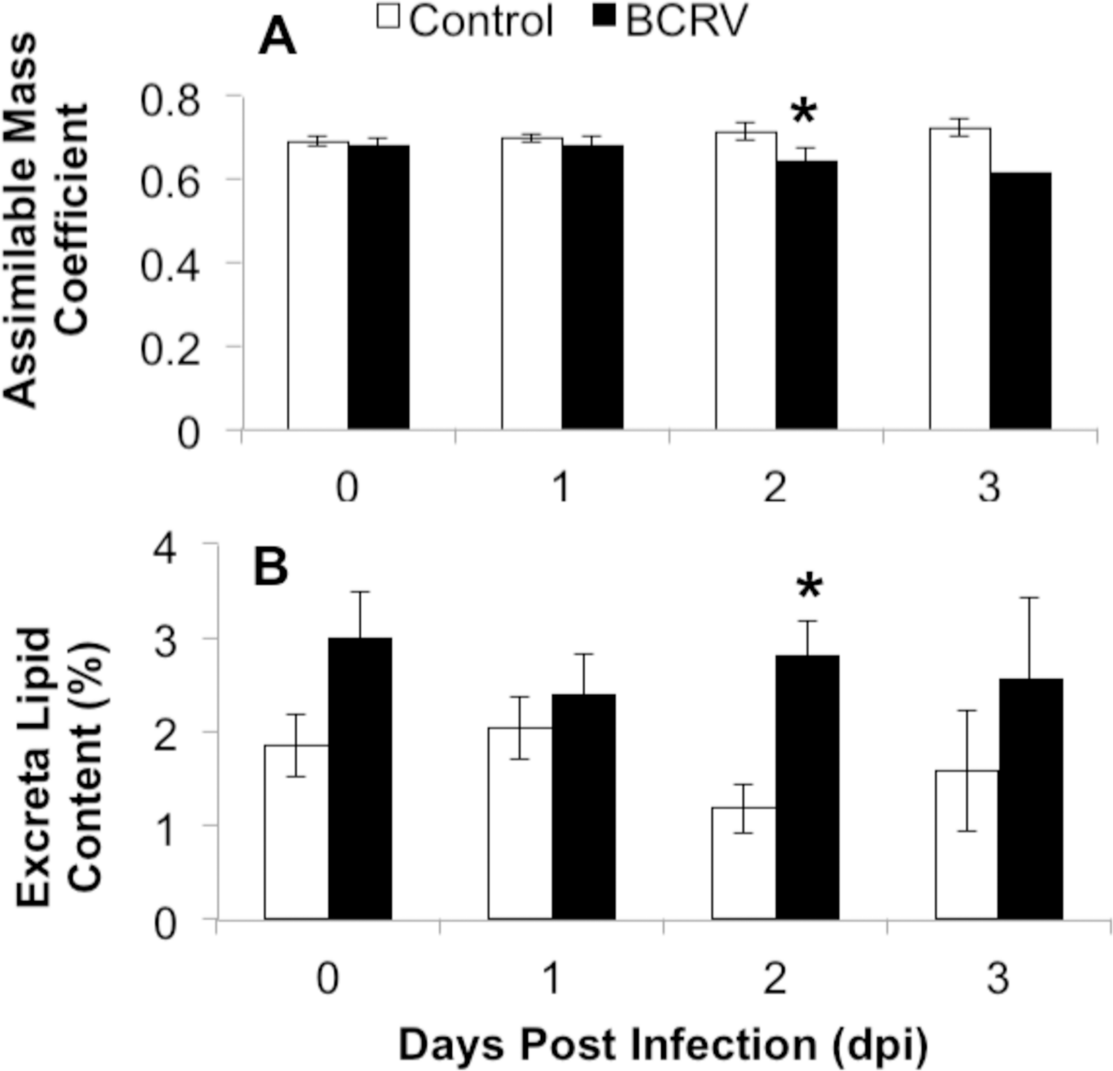
**Apparent assimilable mass coefficient (a) and excreta lipid content (B) during infection (mean ± SEM).** Nestlings were age 7 days post-hatch when infected (0 DPI). n = 11–12 at 0-1DPI; n = 8 at 2DPI; n = 4 at 3DPI. Asterisks above bars indicate significant difference between treatment groups by two-sample t-test (3 DPI was not statistically analyzed due to low sample size).

Excreta were collected daily to measure digestive function over the course of infection. While there were no significant differences in apparent AMC in the early days post-inoculation (0DPI and 1DPI: t_22_<0.93, p>0.364), BCRV-infected nestlings had significantly lower apparent AMC when compared to controls at 2 DPI (t_14_ = 2.26, p = 0.041) (Fig 4A). Additionally, while there were no significant differences in excreta lipid content in the early days post-inoculation (0DPI and 1DPI: t_22_<2.00, p>0.058), BCRV-infected nestlings excreted significantly more lipid at 2 DPI when compared to controls (t_14_ = 3.87, p = 0.002) (Fig 4B). Excreta and AMC measures were not compared at 3 DPI due to low sample size (n = 4 per group).

**Fig 5:**
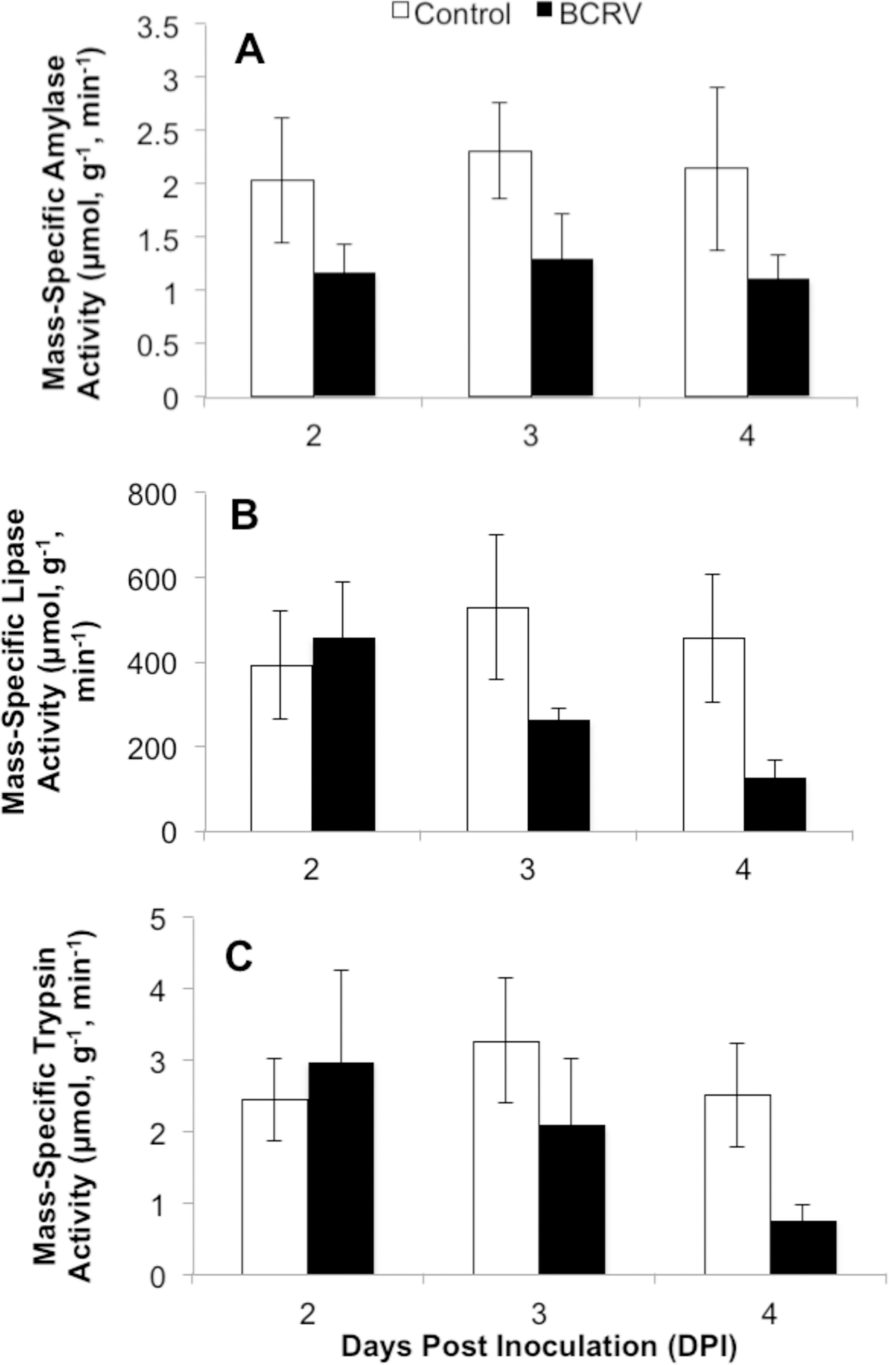
**Pancreatic (A) amylase (B) lipase and (C) trypsin enzyme activities during infection (mean ± SEM).** Nestlings were age 7 days post-hatch when infected (0 DPI). n = 4 per group. Data were analyzed by two-way ANOVA.

**Fig 6:**
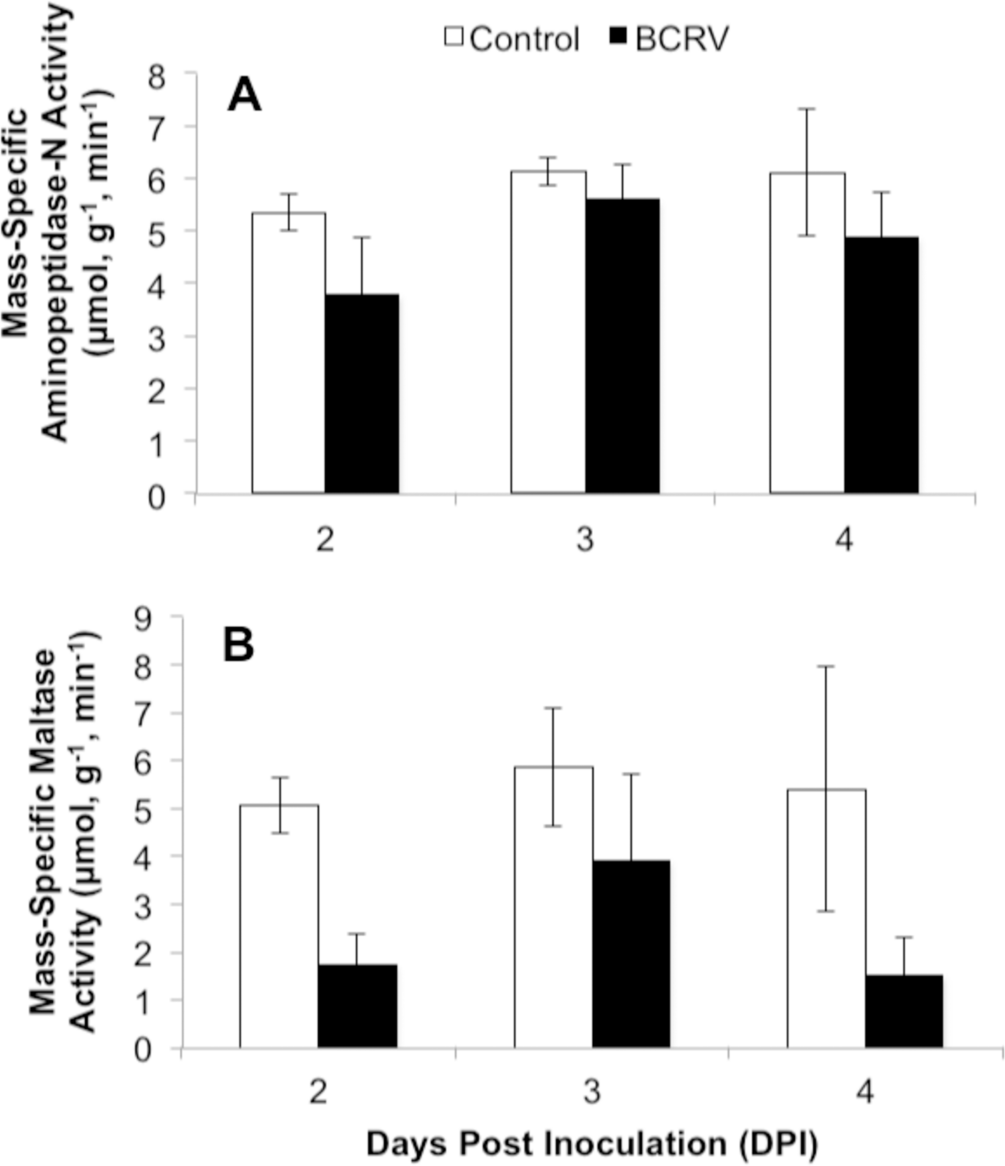
**Intestinal (A) aminopeptidase-N and (B) maltase activities during infection (mean ± SEM).** Nestlings were age 7 days post-hatch when infected (0 DPI). N = 4 per group. Data were analyzed by two-way ANOVA.

Assimilation organs were harvested from cohorts of birds post-mortem at 2, 3, and 4 DPI to examine the impact of BCRV infection of digestive enzyme activity. There were no significant interactions between DPI and treatment group for any measure. Amylase activity per gram of pancreas tissue was significantly lower in BCRV-inoculated birds compared with controls (Treatment F = 7.51, p = 0.013; DPI F = 0.11, p = 0.901) (Fig 5A), yet there were no significant differences in pancreatic lipase or trypsin activities (Treatment F<2.93, p>0.103; DPI F<1.77, p>0.197) (Fig 5B and 5C). Maltase activity per gram of intestinal tissue was significantly lower in BCRV-inoculated birds compared with controls (Treatment F = 9.08, p = 0.007; DPI F = 0.10, p = 0.387) (Fig 6A), yet there were no significant differences in aminopeptidase-N activity (Treatment F = 2.71, p = 0.116; DPI F = 1.01, p = 0.382) (Fig 6B).

## Discussion

Alphaviruses such as Eastern and Western Equine Encephalitis virus, Chikungunya virus, and Sindbis virus are known to cause neuroinvasive disease in humans [57], especially in children and the elderly [28, 58]. The majority of studies performed on age-dependent alphavirus susceptibility have been experimental alphavirus infections of mice or clinical reports of alphavirus infections in humans [31, 32]. We determined the functional impact of an alphavirus infection on growth, maturation, and digestive function in nestling birds. To our knowledge, this is the first study to demonstrate the physiological impact of a viral infection on nestling altricial birds.

Encephalitis is common in neuroinvasive alphavirus disease, but the mechanism of neuroinvasion has not been well studied in any animal model. Chikungunya virus may directly infect endothelial cells of cerebral blood vessels and gain access to the CNS through blood brain barrier disruption, whereas Sindbis virus appears to infect peripheral neurons and gain access to the CNS through axonal transport [57]. However, how these infection pathways change with age and allow for higher rates of neuroinvasion in the young and old is unknown. Additionally, although birds are hosts for at least seven alphaviruses, the pathogenesis of avian alphavirus infections is not well studied [59]. Here we report neuroinvasive disease in nestling house sparrows experimentally infected with BCRV. Encephalitis and high viral load were found in the cerebra of all infected nestlings. No apparent pathology was found outside the central nervous system in the organism, although significant impairment of growth, maturation, and digestive function was observed.

The nestling period of birds is an energetically expensive time during which rapid anatomical growth and maturation occurs. Given that infectious diseases can be costly to an organism in terms of pathological damage, decreased appetite and impaired growth, the nestling period of a bird’s life is likely to be especially susceptible to the impacts of infectious diseases. BCRV-infected birds showed no significant difference in body mass compared with control birds due to a strict meal size matching between treatment groups and force-feeding of infected birds when they were demonstrating morbidity. Despite similar whole body wet masses between treatment groups, significant reductions in head-beak length and tarsus length were observed by 3 days post-inoculation in infected nestlings. Additionally, BCRV infection led to significantly reduced wing chord length in nestlings.

Our findings of reduced tarsus, head-beak, and wing lengths during infection are particularly striking given that numerous studies have shown prioritization of skeletal growth and feather growth relative to mass gain in nestlings faced with acute or chronic food restriction [48, 60, 61, 62, 63, 64]. It has been posited that maintenance of skeletal growth occurs proximally due to strict endocrine control of the skeletal growth program, and that ultimately larger structural size improves competitive abilities and fledging success in altricial birds. Flight ability has also been shown to be prioritized during developmental stress [65, 66, 67]. Wing length is particularly important for fledging and foraging success and predator avoidance post-fledging [63, 68, 69]. Allocation of resources to wing growth can mitigate the negative effects of a small body size on fledging time [44]. In our study BCRV-infection clearly altered a typically well-preserved and adaptive developmental program of skeletal and wing growth in nestlings and this altered growth would likely have negative ecological consequences for any nestlings surviving infection in the wild. Indeed, field studies of natural BCRV infections in house sparrow nestlings have reported the detrimental impacts of BCRV on survival and fledgling success [17, 70, 39]. Specifically, house sparrow nestlings found infected with BCRV at a young age (less than 12 days of age) often exhibit higher viremia, higher mortality, and reduced fledging success compared to those nestlings infected at older ages [71]. The immune system of house sparrows develops during the nestling and post-fledging periods [37, 38]. We hypothesize that younger nestlings are more susceptible to alphavirus neuroinvasion than older nestlings and that this neurological impairment results in impaired growth and physiological functions, further contributing to the poor survival of the infected nestlings.

Nestling development is closely linked to digestive function, and although the timecourse for development of the digestive system and body size and composition in altricial nestlings has been documented under normal conditions and in conditions of altered food availability and quality [47, 48], no studies exist which have examined the development of nestling birds during microparasite infection, or how digestive function is affected during infection. Digestive function declined in our infected birds within two days of infection. Specifically, despite having the same food intake as controls, BCRV-infected nestlings had decreased digestive efficiency (decreased AMC). This decrease in digestive efficiency could result in a reduction of assimilable resources available for skeletal and wing growth. Decreased AMC may partially be due to reduced expression of pancreatic amylase and intestinal maltase enzymes, thereby preventing carbohydrate digestion, absorption, and use in growth processes. Additionally, an increased excretion of ingested lipid and reduced liver lipid content were observed in infected birds. When birds have excess resources, they are stored as fat rather than allocated to growth of excessively large organs [43]; excessive excretion of lipid and reduced enzymatic function indicates that infected birds did not have extra nutritional resources to store. Reduced nutritional resources in infected birds may prevent birds from mounting sufficient immune responses or from repairing damaged tissue that results from viral invasion or inflammation.

## Conclusions

Our work demonstrates that arbovirus infections during a critical period of growth and development in young nestlings impairs digestive function and skeletal and feather growth. This impairment likely contributes to the poor fledging success recorded for BCRV-infected house sparrow nestlings in the wild and exemplifies the complex tradeoffs that can occur between immune system function and other physiological processes during periods of critical energetic needs in an organism’s life. Future work in this area should focus on elucidating the mechanism through which neuroinvasive alphavirus disease impairs growth and development in young animals.

## References

[1] Sheldon BC, Verhulst S. Ecological immunology: costly parasite defenses and trade-offs in evolutionary ecology. Trends Ecol Evol. 1996; 11: 317–321. 21237861 10.1016/0169-5347(96)10039-2

[2] Lochmiller RL, Deerenberg C. Trade-offs in evolutionary immunology: just what is the cost of immunity? Oikos. 2000; 88: 87–98.

[3] Fassbinder-Orth CA. Gene expression quantification methods in ecoimmunology: from qPCR to RNA-Seq. Integr Comp Biol. 2014; 54: 396–406. doi: 10.1093/icb/icu023 24812328 10.1093/icb/icu023

[4] Hawley D.M, Altizer SM. Disease ecology meets ecological immunology: understanding the links between organismal immunity and infection dynamics in natural populations. Funct Ecol. 2011; 25: 48–60.

[5] Viney ME, Riley EM, and Buchanan KL. Optimal immune responses: immunocompetence revisited. Trends Ecol and Evolut. 2005; 20: 665–669. 10.1016/j.tree.2005.10.003 16701455

[6] Owen JP, Nelson AC, and Clayton DH. Ecological immunology of bird-ectoparasite systems. Trends Parasitol. 2010; 26: 530–539. doi: 10.1016/j.pt.2010.06.005 20599426 10.1016/j.pt.2010.06.005

[7] Boughton R, Joop G, Armitage S. Outdoor immunology: methodological considerations for ecologists. Funct Ecol. 2011; 25: 81–100.

[8] Demas GE, Zysling DA, Beechler BR, Muehlenbein MP, French SS. Beyond phytohaemagglutinin: assessing vertebrate immune function across ecological contexts. J Anim Ecol. 2011; 80: 710–30. doi: 10.1111/j.1365-2656.2011.01813.x 21401591 10.1111/j.1365-2656.2011.01813.x

[9] Moreno-García M, Córdoba-Aguilar A, Condé R, Lanz-Mendoza H. Current immunity markers in insect ecological immunology: assumed trade-offs and methodological issues. Bull Entomol Res. 2013; 103: 127–139. doi: 10.1017/S000748531200048X 22929006 10.1017/S000748531200048X

[10] Ezenwa VO, Etienne RS, Luikhart G, Beja-Pereira A, Jolles A. Hidden consequences of living in a wormy world: nematode-induced immune suppression facilitates tuberculosis invasion in African buffalo. Am Nat. 2010; 176: 613–24. doi: 10.1086/656496 20849271 10.1086/656496

[11] Adelman JS, Mayer CM, Hawley DM. Infection reduces anti-predator behaviors in house finches. J Avian Biol. 2017; doi: 10.1111/jav.01058 29242677 10.1111/jav.01058 PMC5724792

[12] Paessler S, Taylor K. Encephalitic Development in Alphaviral Infection. In Non-Flavivirus Encephalitis (Ed. Tkachev S.). InTech 2011; doi: 10.5772/24175

[13] Luers AJ, Adams SD, Smalley JV, Campanella JJ. A phylogenomic study of the genus alphavirus employing whole genome comparison. Comp Funct Genomics. 2005; 6: 217–227. doi: 10.1002/cfg.478 18629194 10.1002/cfg.478 PMC2447488

[14] Suhrbier A, Jaffar-Bandjee MC, Gasque P. Arthritogenic alphaviruses-an overview. Nat Rev Rheumatol. 2012; 8: 420–429. doi: 10.1038/nrrheum.2012.64 22565316 10.1038/nrrheum.2012.64

[15] Adelman ZN, Miller DM, Myels KM. Bed Bugs and Infectious Disease: A Case for the Arboviruses. PLOS Path. 2013; 9: e1003462. 10.1371/journal.ppat.1003462 PMC3744395 23966852

[16] Moore AT, Edwards EA, Brown MB, Komar N, Brown CR. Ecological correlates of Buggy Creek virus infection in *Oeciacus vicarius*, southwestern Nebraska, 2004. J Med Entomol. 2007; 44: 42–49. 17294919 10.1603/0022-2585(2007)44[42:ecobcv]2.0.co;2

[17] Scott TW, Bowen GS, Monath TP. A field study on the effects of Fort Morgan virus, an arbovirus transmitted by swallow bugs, on the reproductive success of cliff swallows and symbiotic house sparrows in Morgan County, Colorado, 1976. Am J Trop Med Hyg. 1984; 33: 981–991. 6091471 10.4269/ajtmh.1984.33.981

[18] O’Brien VA, Meteyer CU, Ip HS, Long RR, Brown CR. Pathology and virus detection in tissues of nestling house sparrows naturally infected with Buggy Creek virus (Togaviridae). J Wildl Dis. 2010; 46: 23–32. doi: 10.7589/0090-3558-46.1.23 20090015 10.7589/0090-3558-46.1.23

[19] Huyvaert KP, Moore AT, Panella NA, Edwards EA, Brown MB, Komar N, et al Experimental inoculation of house sparrows (*Passer domesticus*) with Buggy Creek virus. J Wildl Dis. 2008; 44: 331–340. doi: 10.7589/0090-3558-44.2.331 18436665 10.7589/0090-3558-44.2.331

[20] McLean RG, Crans WJ, Caccamise DF, McNelly J, Kirk LJ, Mitchell CJ, et al Experimental infection of wading birds with eastern equine encephalitis virus. J Wild Dis. 1995; 31: 502–508. 10.7589/0090-3558-31.4.502 8592381

[21] Reisen WK, Chiles RE, Martinez VM, Fang Y, Green EN. Experimental infection of California birds with western equine encephalomyelitis and St. Louis encephalitis viruses. J Med Entomol. 2003; 40: 968–982. 14765678 10.1603/0022-2585-40.6.968

[22] Reisen WK, Martinez VM, Fang Y, Garcia S, Ashtari S, Wheeler SS, et al Role of California (*Callipepla californica*) and Gambel's (*Callipepla gambelii*) quail in the ecology of mosquito-borne encephalitis viruses in California, USA. Vector Borne Zoonotic Dis. 2006; 6: 248–260. doi: 10.1089/vbz.2006.6.248 16989564 10.1089/vbz.2006.6.248

[23] Unnasch RS, Sprenger T, Katholi CR, Cupp EW, Hill GE, Unnasch TR. A dynamic transmission model of eastern equine encephalitis virus. Ecol Model. 2006; 192: 425–440. 10.1016/j.ecolmodel.2005.07.011 PMC1364502 16501661

[24] Graham AL, Allen JE, Read AF. Evolutionary causes and consequences of immunopathology. Annu Rev Ecol Evol Syst. 2005; 36: 373–397.

[25] Sears BF, Rohr JR, Allen JE, Martin LB. The economy of inflammation: when is less more? Trends Parasitol. 2011; 27: 382–387. doi: 10.1016/j.pt.2011.05.004 21680246 10.1016/j.pt.2011.05.004

[26] Koyuncu OO, Hogue IB, Enquist LW. Virus infections in the nervous system. Cell Host Microbe. 2013; 13: 379–393. doi: 10.1016/j.chom.2013.03.010 23601101 10.1016/j.chom.2013.03.010 PMC3647473

[27] Go YY, Balasuriya UBR, Lee C. Zoonotic encephalitides caused by arboviruses: transmission and epidemiology of alphaviruses and flaviviruses. Clin Exp Vaccine Res. 2014; 3: 58–77. doi: 10.7774/cevr.2014.3.1.58 24427764 10.7774/cevr.2014.3.1.58 PMC3890452

[28] Lennette EH, Koprowki H. Influence of age on the susceptibility of mice to infection with certain neurotropic viruses. J Immunol. 1944; 49: 175–191.

[29] Deresiewicz RL, Thaler SJ, Hsu L, Zamani AA. Clinical and neuroradiographic manifestations of eastern equine encephalitis. N Engl J Med. 1997; 336: 1867–74. doi: 10.1056/NEJM199706263362604 9197215 10.1056/NEJM199706263362604

[30] Ludlow M, Kortekaas J, Herden C, Hoffmann B, Tappe D, Trebst C, et al Neurotropic virus infections as the cause of immediate and delayed neuropathology. Acta Neuropathol. 2016; 131: 159–184. doi: 10.1007/s00401-015-1511-3 26659576 10.1007/s00401-015-1511-3 PMC4713712

[31] Tucker PC, Strauss EG, Kuhn RJ, Strauss JH, Griffin DE. Viral determinants of age-dependent virulence of Sindbis virus for mice. J Virol. 1993; 67: 4605–4610. 8392602 10.1128/jvi.67.8.4605-4610.1993 PMC237845

[32] Valero N, Anez F, Larreal Y, Arias J, Rodriguez Z, Espina L. Evaluation of immunity against Venezuelan equine encephalitis virus and dengue in the human population of San Carlos, the Almirante Padilla Island Municipality, Zulia State, Venezuela. 1996. Invest Clin. 2001; 42: 161–169. 11552505

[33] Scott TW, Edman JD, Lorenz LH, Hubbard JL. Effects of disease on vertebrates’ ability behaviorally to repel host-seeking mosquitoes. Misc Publ Entomol Soc Am. 1988; 65: 9–12.

[34] Swayne DE, Beck JR, Smith CS, Shieh WJ, Zaki SR. Fatal encephalitis and myocarditis in young domestic geese (*Anser anser domesticus*) caused by West Nile virus. Emerg Infect Diseases. 2001; 7: 751–753. 11585545 10.3201/eid0704.010429 PMC2631765

[35] Nemeth NM, Bowen RA. Dynamics of passive immunity to West Nile virus in domestic chickens (*Gallus gallus domesticus*). Am J Trop Med Hyg. 2007; 76: 310–317. 17297041

[36] O'Brien VA, Meteyer CU, Reisen WK, Ip HS, Brown CR. Prevalence and pathology of West Nile virus in naturally infected house sparrows, western Nebraska, 2008. Am J Trop Med Hyg. 2010; 82: 937–944. doi: 10.4269/ajtmh.2010.09-0515 20439979 10.4269/ajtmh.2010.09-0515 PMC2861390

[37] Killpack TL, Karasov WH. Ontogeny of adaptive antibody response to a model antigen in captive altricial zebra finches. PLoS ONE. 2012; 7: e47294 doi: 10.1371/journal.pone.0047294 23056621 10.1371/journal.pone.0047294 PMC3467253

[38] Killpack TL, Oguchi Y, Karasov WH. Ontogenetic patterns of constitutive immune parameters in altricial house sparrows. J Avian Biol. 2013; 44: 513–520.

[39] O’Brien VA, Brown CR. Arbovirus infection is a major determinant of fitness in House Sparrows (*Passer domesticus*) that invade Cliff Swallow (*Petrochelidon pyrrhonota*) colonies. Auk. 2012; 129(4): 707–715.

[40] Mireles AJ, Kim SM, Klasing KC. An acute inflammatory response alters bone homeostasis, body composition, and the humoral immune response of broiler chickens. Poult Sci. 2005; 84: 553–560. doi: 10.1093/ps/84.4.553 15844811 10.1093/ps/84.4.553

[41] Iseri VJ, Klasing KC. Dynamics of the systemic components of the chicken (*Gallus gallus domesticus*) immune system following activation by *Escherichia coli*; implications for the costs of immunity. Dev Comp Immunol. 2013; 40: 248–257. doi: 10.1016/j.dci.2013.02.005 23500513 10.1016/j.dci.2013.02.005

[42] Searcy WA, Peters S, Nowicki S. Effects of early nutrition on growth rate and adult size in song sparrows, *Melospiza melodia*. J Avian Biol. 2004; 35: 269–279.

[43] Takenaka M, Niizuma Y, Watanuki Y. Resource allocation in fledglings of the rhinoceros auklet under different feeding conditions: an experiment manipulating meal size and frequency. Can J Zool. 2005; 83: 1476–1485.

[44] Miller DA. Morphological plasticity reduces the effect of poor developmental conditions on fledging age in mourning doves. Proc R Soc B. 2010; 277: 1659–1665. doi: 10.1098/rspb.2010.0022 20129984 10.1098/rspb.2010.0022 PMC2871865

[45] Bosman DS, Stienen EWM, Lens L. Sex, growth rate, rank order after brood reduction, and hatching date affect first-year survival of long-lived Herring Gulls. J Field Ornithol. 2016; 87: 391–403.

[46] Naef-Danzer B, Gruebler MU. Post-fledging survival of altricial birds: ecological determinants and adaptation. J Field Ornithol. 2016; 87: 227–250.

[47] Lepczyk CA, Caviedes-Vidal E, Karasov WH. Digestive responses during food restriction and realimentation in nestling house sparrows (*Passer domesticus*). Physiol Zool. 1998; 71: 561–573. 9754533 10.1086/515965

[48] Killpack TL, Karasov WH. Growth and development of house sparrows (*Passer domesticus*) in response to chronic food restriction throughout the nestling period. J Exp Biol. 2012; 215: 1806–1815. doi: 10.1242/jeb.066316 22573759 10.1242/jeb.066316

[49] Seel DC. Food, feeding rates and body temperature in the nestling house sparrow, *Passer domesticus*, at Oxford. Ibis. 1969; 111: 36–47.

[50] Caviedes-Vidal E, Karasov WH. Developmental changes in digestive physiology of nestling house sparrows, *Passer domesticus*. Physiol Biochem Zool. 2001; 74: 769–782. doi: 10.1086/322966 11517462 10.1086/322966

[51] Fassbinder-Orth CA, Barak V, Brown C. Immune Responses of a Native and an Invasive Bird to Buggy Creek Virus (*Togavirida*e: Alphavirus) and its Arthropod Vector, the Swallow Bug (*Oeciacus vicarius*). PLOS One. 2013; 8: e58045 doi: 10.1371/journal.pone.0058045 23460922 10.1371/journal.pone.0058045 PMC3584039

[52] Fassbinder-Orth CA, Karasov WH. Effects of feed restriction and realimentation on digestive and immune function in the leghorn chick. Poult Sci. 2006; 85: 1449–1456. doi: 10.1093/ps/85.8.1449 16903477 10.1093/ps/85.8.1449

[53] National Research Council. Nutritional Energetics of Domestic Animals and Glossary of Energy Terms. Natl. Acad. Sci. 1981; Washington, DC.

[54] Miller MR, Reinecke KJ. Proper expression of metabolizable energy in avian energetics. Condor. 1984; 86: 396–400.

[55] Dahlqvist A. Assay of intestinal disaccaridases. Scand J Clin Lab Invest. 1984; 44: 69–72. 10.3109/00365518409161400 6719024

[56] Martınez del Rio C, Brugger KE, Rios JL, Vergara ME, Witmer M. An experimental and comparative study of dietary modulation of intestinal enzymes in European starlings (*Sturnus vulgaris*). Physiol Zool. 1995; 68: 490–511.

[57] Passoni G, Langevin C, Palha N, Mounce BC, Briolat V, Affaticati P, et al Imaging of viral neuroinvasion in the zebrafish reveals that Sindbis and chikungunya viruses favour different entry routes. Dis Models Mech. 2017; 10: 847–857. 10.1242/dmm.029231 PMC5536907 28483796

[58] Calisher CH. Medically important arboviruses of the United States and Canada. Clin Microbiol Rev. 1994; 7: 89–116. 8118792 10.1128/cmr.7.1.89 PMC358307

[59] Fassbinder-Orth CA, Barak VA, Rainwater EL, Altrichter AM. Buggy Creek virus (*Togaviridae*: *Alphavirus*) upregulates expression of PRRs and interferons in house sparrows (*Passer domesticus*). Vector-Borne and Zoonotic Dis. 2014; 14: 439–445. 10.1089/vbz.2013.1531 PMC4050711 24866749

[60] Lacombe D, Bird DM, Hibbard KA. Influence of reduced food availability on growth of captive American kestrels. Can J Zool. 1994; 72: 2084–2089.

[61] Negro JJ, Chastin A, Bird DM. Effects of Short-Term Food Deprivation on Growth of Hand-Reared American Kestrels. Condor. 1994; 96: 749–760.

[62] Lepczyk CA, Karasov WH. Effect of ephemeral food restriction on growth of house sparrows. Auk. 2000; 117: 164–174.

[63] Dahdul WM, Horn MH. Energy allocation and postnatal growth in captive elegant tern (*Sterna elegans*) chicks: responses to high-versus low-energy diets. Auk. 2003; 120: 1069.

[64] Moe B, Brunvoll S, Mork D, Brobakk TE, Bech C. Developmental plasticity of physiology and morphology in diet-restricted European shag nestlings (Phalacrocorax aristotelis). J Exp Biol. 2004; 207: 4067–4076. doi: 10.1242/jeb.01226 15498952 10.1242/jeb.01226

[65] Bize P, Roulin A, Bersier LF, Pfluger D, Richner H. Parasitism and developmental plasticity in Alpine swift nestlings. J Anim Ecol. 2003; 72: 633–639. 10.1046/j.1365-2656.2003.00734.x 30893964

[66] Chin EH, Love OP, Verspoor JJ, Williams TD, Rowley K, Burness G. Juveniles exposed to embryonic corticosterone have enhanced flight performance. Proc R Soc B. 2009; 276: 499–505. doi: 10.1098/rspb.2008.1294 18842541 10.1098/rspb.2008.1294 PMC2664354

[67] Miller DA. Immediate and delayed effects of poor developmental conditions on growth and flight ability of juvenile mourning doves Zenaida macroura. J Avian Biol. 2011; 42: 151–158.

[68] Reid K, Prince PA, Croxall JP. Fly or die: the role of fat stores in the growth and development of Grey-headed Albatross *Diomedea chrysostoma* chicks. Ibis. 2000; 142: 188–198.

[69] Deguchi T, Takahashi A, Watanuki Y. Proximate Factors Determining Age and Mass at Fledging in Rhinoceros Auklets (*Cerorhinca monocerata*): Intra- and Interyear Variations. Auk. 2004; 121: 452–462.

[70] Hayes RO, Francy DB, Lazuick JS, Smith GC, Gibbs EPJ. Role of the cliff swallow bug (*Oeciacus vicarius*) in the natural cycle of a western equine encephalitis-related alphavirus. J Med Entomol. 1977; 14: 257–262.

[71] O’Brien VA, Brown CR. Seasonal variation and age-related correlates of Buggy Creek virus (Togaviridae) infection in nestling house sparrows. J Wildl Dis. 2012; 48: 138–147. doi: 10.7589/0090-3558-48.1.138 22247382 10.7589/0090-3558-48.1.138

